# Development of a nomogram for predicting the presence of combined pulmonary fibrosis and emphysema

**DOI:** 10.1186/s12890-021-01725-x

**Published:** 2021-11-07

**Authors:** Xueting Yuan, Jin Jin, Xiaomao Xu

**Affiliations:** 1 The Key Laboratory of Geriatrics, Beijing Institute of Geriatrics, Beijing Hospital, National Center of Gerontology, National Health Commission, Institute of Geriatric Medicine, Chinese Academy of Medical Sciences, No. 1 DaHua Road, Dong Dan, Beijing, 100730 People’s Republic of China; 2 Department of Pulmonary and Critical Care Medicine, Beijing Hospital, National Center of Gerontology, Institute of Geriatric Medicine, Chinese Academy of Medical Sciences, Beijing, People’s Republic of China

**Keywords:** CPFE, IPF, Nomogram, Risk factors, Multivariable logistic regression analysis

## Abstract

**Background:**

In the clinical management of patients with combined pulmonary fibrosis and emphysema (CPFE), early recognition and appropriate treatment is essential. This study was designed to develop an accurate prognostic nomogram model to predict the presence of CPFE.

**Methods:**

We retrospectively enrolled 85 patients with CPFE and 128 patients with idiopathic pulmonary fibrosis (IPF) between January 2015 and January 2020. Clinical characteristics were compared between groups. A multivariable logistic regression analysis was performed to identify risk factors for CPFE. Then, and a nomogram to predict the presence of CPFE was constructed for clinical use. Concordance index (C-index), area under the receiver operating characteristic curve (AUC), and calibration plot was used to evaluate the efficiency of the nomogram.

**Results:**

Compared to the IPF group, the proportion of patients with male, smoking and allergies were significantly higher in the CPFE group. In terms of pulmonary function tests, patients with CPFE had lower FEV1/FVC%, DLCO/VA% pred, and higher RV, RV%pred, VC, VC%pred, TLC%pred, VA, TLC, TLC%pred, FVC, FVC%pred and FEV1 with significant difference than the other group. Positive correlation was found between DLCO and VA%, RV%, TLC% in patients with IPF but not in patients with CPFE. By multivariate analysis, male, smoking, allergies, FEV1/FVC% and DLCO/VA%pred were identified as independent predictors of the presence of CPFE. The nomogram was then developed using these five variables. After 1000 internal validations of bootstrap resampling, the C-index of the nomogram was 0.863 (95% CI 0.795–0.931) and the AUC was 0.839 (95% CI 0.764–0.913). Moreover, the calibration plot showed good concordance of incidence of CPFE between nomogram prediction and actual observation (Hosmer–Lemeshow test: *P* = 0.307).

**Conclusions:**

Patients of CPFE have a characteristic lung function profile including relatively preserved lung volumes and ventilating function, contrasting with a disproportionate reduction of carbon monoxide transfer. By incorporating clinical risk factors, we created a nomogram to predict the presence of CPFE, which may serve as a potential tool to guide personalized treatment.

## Background

Pulmonary interstitial fibrosis and emphysema have long been perceived as two separate diseases. Interstitial lung diseases (ILD) encompass a large and heterogeneous group of diffuse parenchymal lung disorders characterized by distinct forms and severity of inflammation and fibrosis in alveolar walls and cavities [1]. Idiopathic pulmonary fibrosis (IPF), an ILD of unknown cause is invariably progressive and associated with poor prognosis [2]. Emphysema, most often caused by long-term exposure to cigarette smoke, featured with abnormal and permanent enlargement of air spaces distal to the terminal bronchioles, is one of the major pathobiological processes leading to chronic obstructive pulmonary disease [3]. Progressive inflammation damages the airway mucosal epithelium, which in turn leads to airflow limitation and lung parenchymal destruction [4]. Therefore, emphysema and fibrosis are often considered distinct entities with unique pathophysiologic manifestations, but in the past 15 years, there has been an increasing recognition that these two processes may coexist in individual patients. “Combined pulmonary fibrosis and emphysema (CPFE)” was first described as a well-defined syndrome by Cottin et al. in 2005 [5].

Patients with CPFE are characterized by a relatively normal lung function due to the counterbalancing effects of fibrotic (restrictive factor) and emphysematous (obstructive factor) components [6, 7], which often lead to underestimating the severity of CPFE, or even a delayed or missed clinical diagnosis. A previous study conducted by Mejía M et al. reported that in the series of the 110 patients initially diagnosed with IPF, 28% were reevaluated and classified as CPFE [8]. The other study found that CPFE was found in 33.5% of 660 patients with usual interstitial pneumonia (UIP) [9]. Although computed tomography (CT) scan of the chest is routinely performed in patients with IPF, the development of emphysema is considered as a long cumulative process, which gas exchange and mechanical abnormalities may predate radiographic low attenuation areas of the lung parenchyma. Consequently, the presence of CPFE in patients diagnosed with IPF is of concern. In addition, CPFE is frequently complicated by pulmonary hypertension [10], lung cancer [11], acute exacerbations [12], and leading to poor natural history and prognosis [13]. Currently, there is still a lack of specific drugs for clinical treatment [14]. Considering this, a predictive model with reliable efficacy is of great importance to helps us raise the profile of patients with possible CPFE early (e.g., before imaging, or some who refused frequent CT scans), so as to conduct appropriate clinical treatment of CPFE.

The nomogram provides a visualization of the regression equation, which has been accepted as a reliable tool to create a simple intuitive graph of a statistical predictive model that quantifies the risk of a clinical event [15]. In this work, we performed a retrospective study to create an easy-to-use risk assessment nomogram model integrating multiple clinical risk factors for predicting the presence of CPFE to support clinicians in their treatment recommendations.

## Methods

### Patient participants

This retrospective study involved 85 patients with CPFE and 128 patients with IPF during the period between January 2015 to January 2020, from Beijing Hospital. Diagnosis of IPF was made according to an official ATS/ERS/JRS/ALAT guideline [16]: subpleural, basal, predominantly reticular abnormality or honeycombing, with or without traction bronchiectasis, and the absence of an inconsistent UIP pattern. CPFE was defined according to Cottin et al.'s definitions [5], namely the presence of classic features of centrilobular and/or paraseptal emphysemas (≥ 10%) in the upper lobes and pulmonary fibrosis (mainly IPF/UIP) in the lower lobes radiographically. Patients with other specific types of ILD, such as, pneumoconiosis, hypersensitivity pneumonitis, sarcoidosis, pulmonary Langerhans cell histiocytosis, lymphangioleiomyomatosis or eosinophilic pneumonias were excluded. The study was approved by Ethics Committee of Beijing Hospital (2020BJYYEC-053-02). Written informed consent was obtained from all participants.

### Data collection

The following demographic and clinical data were extracted from electronic medical records at the time of the initial high-resolution computed tomography (HRCT) of the chest study: population characteristics (age, gender, body mass index (BMI), smoking history, thoracic operation history, allergies (drug allergy, with or without food allergy and hay fever), and occupational dust exposure), comorbidities (hypertension, reflux esophagitis, coronary disease, osteoporosis, stroke, and tumor), pulmonary function tests (RV, RV%pred, VC, VC%pred, VA, VA%pred, TLC, TLC%pred, FVC, FVC%pred, FEV1/FVC%, FEV1, FEV1%pred, DLCO, DLCO/VA, and DLCO/VA%pred), data required for the Charlson Comorbidity Index (CCI) [17], and composite physiologic index (CPI) [18]. Spirometric data were collected using MasterScreen™ spirometer (CareFusion, Germany, 234 Gmbh) and the European Community of Coal and Steel (ECCS) predicted equations were used to calculate predicted values [19]. These data were verified by two experienced physicians independently.

### Statistical analysis

Continuous variables were described using median and interquartile range (IQR). Categorical variables were described as number (%). Non-normal distributed continuous data were compared using Mann–Whitney-U test. Categorical data were compared using X^2^ test or the Fisher exact test. Correlations between variables were analyzed using the Spearman's rank correlation. Correlation strength was selected by an absolute correlation (|r|> 0.2) and the selected correlation was plotted as an undirected network graph. Multivariable logistic regression analysis was implemented to identify the powerful combination of significant factors which were utilized to build a prediction model and a nomogram was used to visualize the model.

The nomogram was subjected to 1000 bootstrap resamples for internal validation and the performance was assessed by discrimination and calibration [20]. Harrell’s concordance index (C-index), the area under the receiver operating characteristic curve (AUC) was used to verify the discrimination of the model, while the calibration plot was used to graphically evaluate the calibration of the nomogram. The C-index ranges from 0.5 to 1.0, with 0.5 indicating random chance and 1.0 demonstrating perfect discrimination. In general, an AUC > 0.75 was considered to be relatively good discrimination. Moreover, the Hosmer–Lemeshow (H–L) test was used to examine how well the percentage of the observed probability matched the percentage of predicted probability over deciles of predicted risk.

The statistical analyses were performed with IBM SPSS 25.0 and R 3.4.3 with the rms statistical packages for all the analyses. All tests were 2-sides, and a P value < 0.05 was considered statistically significant.

## Results

### Population characteristics

**Fig. 1:**
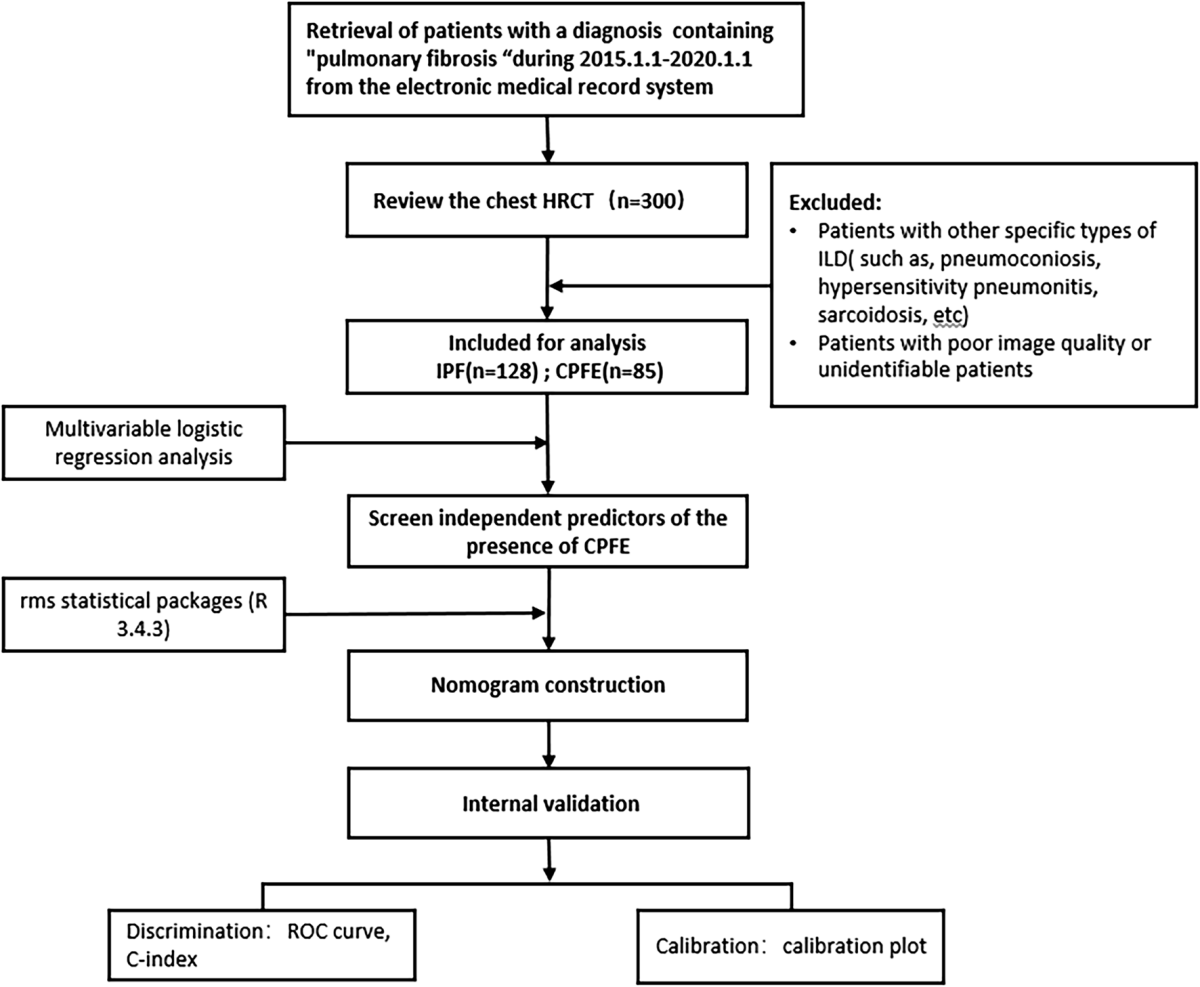
Study flow. *Three indicators (gender (males), smoking, allergies) with statistically significant differences (*P* < 0.05) in the results of the univariate analysis, as well as two key pulmonary function index (FEV1/FVC, DLCO/VA%pred), were included in the multivariable logistic regression analysis to identity independent risk factors

Of the 300 patients initially retrieved from the medical record system, 85 CPFE patients and 128 IPF patients were eventually involved for analysis (Fig. 1). Table 1 summarized the details of baseline characteristics of enrolled patients. Compared to the IPF group, the proportion of patients with male (91.8% vs. 55.5%, *P* = 0.000), smoking (88.1% vs. 46.3%, *P* = 0.000) and allergies (24.4% vs. 11.5%, *P* = 0.000) were significantly higher in the CPFE group. No statistical difference was found in terms of age, BMI, most personal history and comorbidities between two groups (*P* > 0.05). In addition, according to the CCI and CPI assessment, the differences of indexes were not statistically significant (*P* > 0.05) (Table 1).

**Table 1:** Demographic and baseline characteristics of enrolled patients

	All patients (N = 213)	CPFE (N = 85)	IPF (N = 128)	*P* value
Demographic				
Age, median (IQR), yrs	75 (65–81)	75 (66–81)	75 (64–82)	0.72
Gender, Male, n (%)	149 (70.0)	78 (91.8)	71 (55.5)	0.000*
BMI (kg/m^2^)	24.9 (22.3–26.7)	24.7 (21.1–26.6)	25.0 (22.6–27.4)	0.32
BMI ≥ 24, n (%)	29 (17.1)	11 (15.5)	18 (18.2)	0.65
Smoking, n (%)	131 (63.3)	74 (88.1)	57 (46.3)	0.000*
Thoracic operation history, n (%)	6 (2.9)	1 (1.2)	5 (4.1)	0.233
Allergies n (%)	34 (16.7)	20 (24.4)	14 (11.5)	0.015*
Occupational dust exposure, n (%)	45 (22.2)	16 (19.8)	29 (23.8)	0.5
Comorbidities, n (%)				
Hypertension	85 (41.9)	33 (41.3)	52 (42.3)	0.885
Reflux esophagitis	39 (18.9)	17 (20.2)	22 (18.0)	0.691
Coronary disease	65 (31.9)	27 (33.3)	38 (30.9)	0.715
Diabetes	50 (24.4)	19 (23.5)	31 (25.0)	0.801
Osteoporosis	22 (10.8)	7 (8.5)	15 (12.3)	0.396
Chronic kidney diseases	15 (7.4)	4 (4.9)	11 (9.1)	0.270
Stroke	33 (16.0)	14 (17.3)	19 (15.2)	0.690
Tumor	39 (18.3)	18 (21.1)	21 (16.4)	0.378
CCI, median (IQR)	1 (0–2)	1 (0–2)	1 (0–2)	0.80
CPI, median (IQR)	39.4 (30.2–52.3)	38.9 (26.9–53.9)	40.8 (31.9–50.0)	0.73

*P < 0.05

### Comparison and correlation network analysis of pulmonary function indexes

**Fig. 2:**
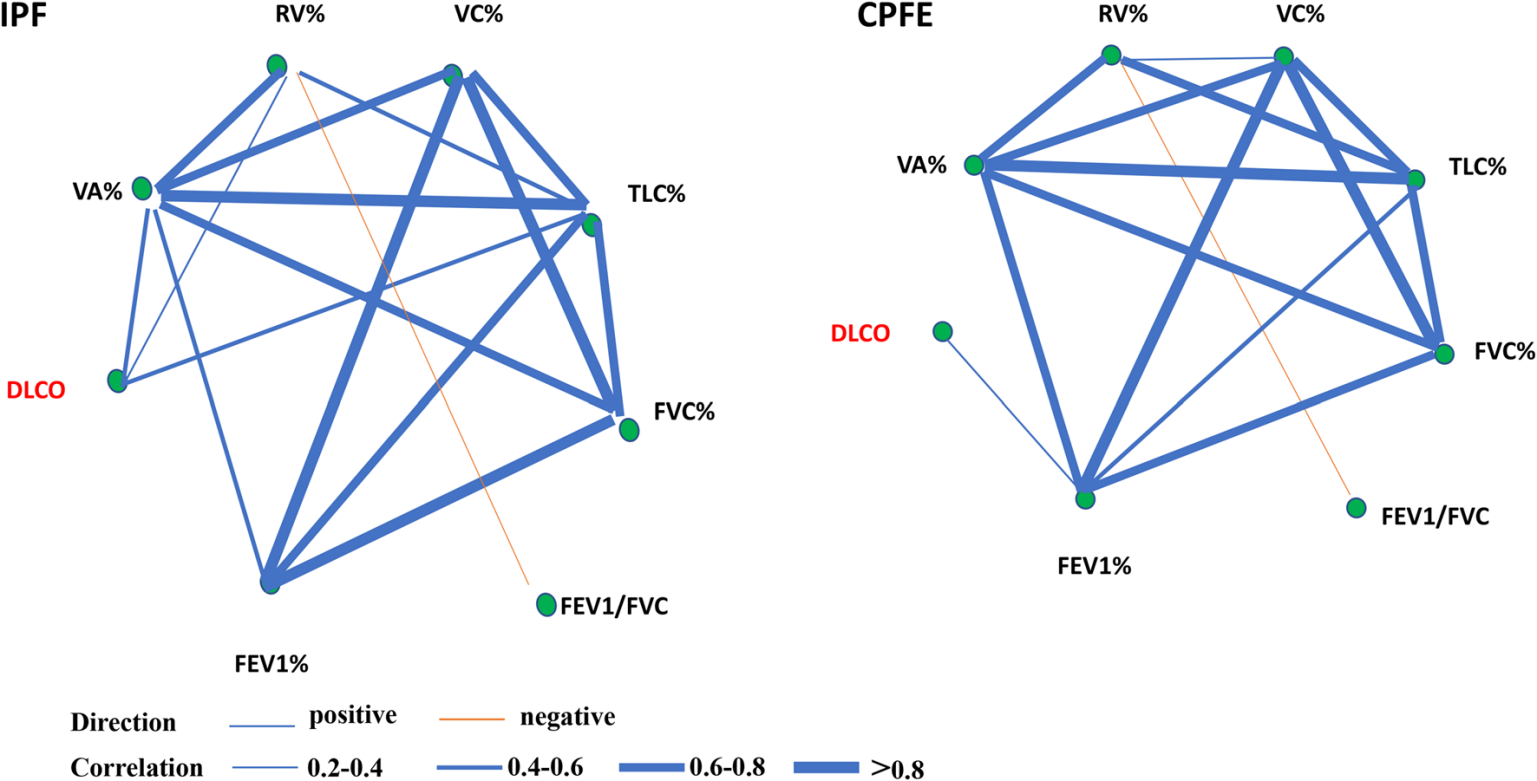
Correlation networks for pulmonary function index among groups. Networks showed different profiles of correlations in CPFE and IPF patients. The width of the edge is proportional to the absolute value of correlation strength (|r|). Edges were shown only when |r|> 0.2. A blue edge indicates a positive correlation, and an orange edge indicates a negative correlation

Patients were admitted primarily for identifying the causes and confirming diagnosis, and pulmonary function was measured as physically permissible for medical purpose. FEV1/FVC% and DLCO/VA in CPFE group were significantly lower than those in IPF group (*P* < 0.05). Conversely, RV, RV%pred, VC, VC%pred, VA, TLC, TLC%pred, FVC, FVC%pred and FEV1 were significantly higher than those in IPF group (*P* < 0.05) (Table 2). Moreover, the correlation analysis between each of the two indexes of pulmonary function was shown as an undirected network graph (Fig. 2). The line's thickness is proportional to the absolute value of correlation strength. DLCO positively correlated with VA% (*r* = 0.470, *P* = 0.000), RV% (*r* = 0.332, *P* = 0.005) and TLC% (*r* = 0.511, *P* = 0.000) in IPF group but no correlation was observed in CPFE group.

**Table 2:** Comparison of pulmonary function indexes among groups

Pulmonary function indexes (IQR)	All patients (N = 213)	CPFE (N = 85)	IPF (N = 128)	*P* value
RV (L)	1.9 (1.6–2.3)	2.1 (1.8–2.5)	1.7 (1.4–2.1)	0.000*
RV%pred	80.3 (69.8–93.5)	82.2 (74.5–99.0)	79.1 (65.2–90.5)	0.042*
VC (L)	2.5 (1.9–3.0)	2.9 (2.3–3.3)	2.1 (1.6–2.7)	0.000*
VC%pred	76.3 (66.7–87.2)	81 (70.1–92.0)	72.8 (64.2–85.1)	0.028*
VA (L)	4.1 (3.2–5.0)	4.8 (3.9–5.3)	3.9 (2.8–4.7)	0.000*
VA%pred	72.3 (63.1–82.3)	76.4 (65.5–83.3)	70.0 (59.8–79.1)	0.118
TLC (L)	4.3 (3.4–5.1)	5 (4.1–5.5)	3.9 (3.1–4.9)	0.000*
TLC%pred	73.3 (65.1–80.6)	77.8 (69.3–84.5)	70.2 (61.0–77.8)	0.002*
FVC (L)	2.4 (1.8–3.0)	2.9 (2.3–3.2)	2.1 (1.6–2.7)	0.000*
FVC%pred	78.1 (67.8–88.4)	81.5 (71.4–93.0)	74.4 (65.5–83.1)	0.013*
FEV1 (L)	1.9 (1.4–2.3)	2.1 (1.7–2.5)	1.7 (1.3–2.2)	0.001*
FEV1%pred	79.0 (66.9–93.6)	79.2 (69.3–91.1)	79.0 (65–94.8)	0.624
FEV1/FVC (%)	82.1 (76.0–87.0)	79.2 (71.5–84.1)	84.4 (78.6–88.5)	0.000*
DLCO/VA (mol/min/kPa/L)	1.2 (0.8–1.5)	1.1 (0.8–1.4)	1.3 (1.0–1.5)	0.021*
DLCO/VA%pred	78.05 (63.35–97.75)	76.3 (59.5–94.1)	86.3 (69.6–98.5)	0.066

*P < 0.05

### Analysis of risk factors for CPFE

The variables univariately associated with CPFE at *P* < 0.05 level, including gender (male), smoking, allergies and key pulmonary function index (FEV1/FVC%, DLCO/VA%pred), were entered into the multivariable logistic regression analysis. As shown in the Table 3, the final multivariate logistic regression analysis yielded five statistically significant independent factors: gender (male) (*P* = 0.025), smoking (*P* = 0.044), allergies (*P* = 0.006), FEV1/FVC% (*P* = 0.003), DLCO/VA% pred (*P* = 0.017).

**Table 3:** Multivariable logistic regression analysis results for presence of CPFE

Variables	B	SE	Waldχ2	P	OR	95% CI
Gender (males)	1.767	0.788	5.021	0.025*	5.852	1.248–27.439
Smoking	1.471	0.732	4.042	0.044*	4.353	1.038–18.262
Allergies	1.824	0.662	7.585	0.006*	6.196	1.692–22.687
FEV1/FVC%	− 0.079	0.027	8.851	0.003*	0.924	0.877–0.973
DLCO/VA%pred	− 0.024	0.010	5.712	0.017*	0.976	0.957–0.996

*P < 0.05

### Nomogram construction and validation

**Fig. 3:**
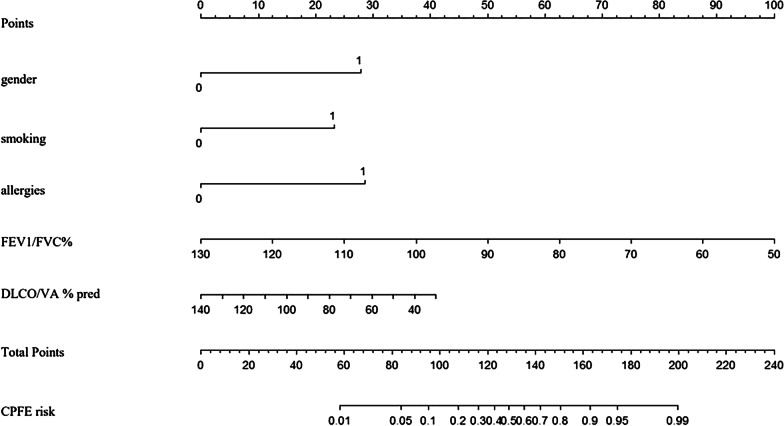
Nomogram of predicting the presence of combined pulmonary fibrosis and emphysema. Instructions for physicians: locate the gender (male) on the gender axis. Draw a line straight upward to the points axis to determine the number of points for the gender. Repeat the process for each of the remaining axes. Sum the points for each of the predictors. Locate the final sum on the total points axis. Draw a line straight down to find the probability of CPFE

The nomogram was constructed based on five independent variables (gender (male), smoking, allergies, FEV1/FVC%, and DLCO/VA% pred) (Fig. 3). Each variable was scored on a scale, and the range of the total points was 0–240. Points for gender (male), smoking, allergies, were 27.6, 23.0 and 28.4 respectively, and the specific points for FEV1/FVC% and DLCO/VA% pred are determined by drawing a line straight upward to the point axis based on the values of the continuous variables. Finally, the total points on the risk axis represents the probability of CPFE.

**Fig. 4:**
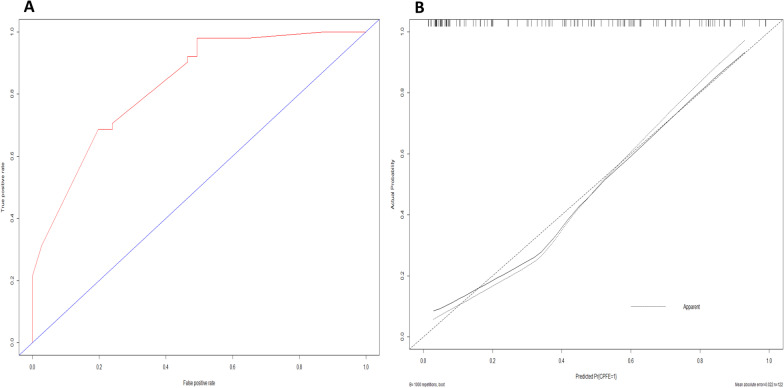
Validation of the nomogram to predict probability of the presence of CPFE. **a** Discrimination. Area under the receiver operating characteristic curve (AUC) is 0.839 (95% CI 0.764–0.913). **b** Calibration plot of the nomogram. The horizontal axis represents the predicted probability and the vertical axis represents the actual probability. Perfect prediction would correspond to the 45° broken line. The dotted and solid lines indicate the observed (apparent) nomogram performance before and after bootstrapping (Hosmer–Lemeshow test: *P* = 0.307)

Performance of this nomogram was assessed by C-index, AUC and calibration plots. The C-index of the nomogram was 0.863 (95% CI 0.795–0.931) and the AUC was 0.839 (95% CI 0.764–0.913), both indicating stable and favorable performance of the model. Moreover, the calibration plot showed good concordance of incidence of CPFE between nomogram prediction and actual observation (Hosmer–Lemeshow test: *P* = 0.307) (Fig. 4).

## Discussion

CPFE is considered as a not fully recognized syndrome characterized by chronic, progressive disease with worsening respiratory symptoms, reduced lung function and poor prognosis [5]. The analysis of risk factors has guiding significance for the early recognition, clinical diagnosis and appropriate treatment. Here, this study describes the clinical characteristics and incorporates multiple clinical variables into a user-friendly nomogram for predicting the presence of CPFE.

With the presence of fibrosis and emphysema concomitantly, pulmonary function tests of CPFE patients are characterized by the preservation of lung volumes and markedly impaired carbon monoxide diffusing capacity, rather than the simple coexistence [21]. The relatively normal lung volumes in CPFE usually result from the counterbalancing effects of the restrictive disorder of fibrosis and the hyperinflation of emphysema [22], meanwhile, the presence of these two factors leading to severe reduction in the amounts of functional alveolar-capillary units [23]. Our results indicated that CPFE patients showed significantly higher lung volume (RV, VC, and TLC) and ventilation indicators (VA, FVC, and FEV1), and DLCO/VA were much more decreased, consistent with previous studies. Moreover, positive correlation was observed between DLCO and VA%, RV%, TLC% in patients with IPF but not in patients with CPFE, which remind us more clinical attention on the variation consistency of lung volume, ventilation and diffusion function indicators.

Most patients with CPFE are males, and they are either current or ex-smokers [24, 25]. Current study demonstrated that smoking has been considered as a risk factor for the development of CPFE [26]. The results of this study came to the same conclusion. The mechanism behind this could also be due to a sequence of events that first cause bronchial inflammation, small airway stenosis and alveolar rupture leading to emphysema; and then additionally stimulate the epithelial mesenchymal transition (EMT) to promote the differentiation of fibroblasts into myofibroblasts [25]. In addition, late-onset increased gastroesophageal reflux (GER) triggered by smoking may also aggravate the fibrotic changes [27].

The higher proportion of patients with allergies in the CPFE group indicated that immune mediators (mast cells, basophils, eosinophils, cytokines, chemokines, etc.) may be associated with the development of disease. Several studies have demonstrated that air contamination is closely related to emphysema and pulmonary fibrosis [28]. Allergy-prone patients who have more abundant and expressed IgE and FcεR receptors [29] may experience more significant inflammation and immune responses when exposed to airborne antigens. Furthermore, TGF-β1, for example, is known to play an major role in the differentiation of fibroblasts into myofibroblasts [30] and eosinophil-derived IL-13 is closely associated with emphysema [31]. It can be speculated that immune impairment in CPFE patients may be more pronounced due to the superimposed effect of immune damage in pulmonary fibrosis and emphysema.

Smoking is undoubtedly a main factor, but not all CPFE patients in this study had a history of smoking (88%). On the one hand, patients with second-hand smoke who are not easily defined may be ignored; on the other hand, it is currently believed that multiple factors are involved in the development of CPFE. Besides inflammation, gene-mediated alveolar damage processes may also lead to CPFE [29]. Oxidative stress and accelerated lung aging with telomere shortening has been proposed as a possible mechanism related to CPFE pathogenesis as well [32]. Another theory suggests that fibrosis occurs predominantly at the base of the lung and can cause local lobe contraction, with progressive compensatory emphysematous changes in the upper lobes of the lung affected by tensile forces [33], consistent with the classical imaging features of CPFE. In short, further studies are needed to shed light on the pathogenesis of CPFE.

In our study, we use multivariable logistic regression analysis to identify significant factors associated with CPFE. Consequently, gender (male), smoking, allergies, FEV1/FVC%, DLCO/VA% pred were identified and used to develop the prognostic nomogram. This nomogram demonstrated good discrimination as assessed by the C-index, AUC value and calibration plot indicating good performance. Nomogram models are used to assess the risks associated with CPFE and they also provide a reference for the clinical management.

Although our study lies in the intuitive characteristics of the disease based on the real-world data and the relatively complete information, which can ensure the accuracy of the model, there are still some limitations. First, the data for the nomogram were retrospectively derived from a single center and may suffer from selection bias. Second, only internal verification was performed and the results may overestimate the effectiveness of the model. Thus, external verification will be optimal for further investigation.

In conclusion, our nomogram incorporating several important clinical variables into the estimate of the risk of CPFE may serve as a potential tool to help inform decision-making by physicians and patients.

## Data Availability

The data that support the findings of this study are available from the corresponding author upon reasonable request.

## Abbreviations

CPFE: Combined pulmonary fibrosis and emphysema
IPF: Idiopathic pulmonary fibrosis
ILD: Interstitial lung diseases
UIP: Usual interstitial pneumonia
BMI: Body mass index
CCI: Charlson Comorbidity Index
CPI: Composite physiologic index
C-index: Concordance index
AUC: Areas under the receiver operating characteristic curve
EMT: Epithelial mesenchymal transition
GER: Gastroesophageal reflux
